# A Physical Activity and Diet Program Delivered by Artificially Intelligent Virtual Health Coach: Proof-of-Concept Study

**DOI:** 10.2196/17558

**Published:** 2020-07-10

**Authors:** Carol Ann Maher, Courtney Rose Davis, Rachel Grace Curtis, Camille Elizabeth Short, Karen Joy Murphy

**Affiliations:** 1 Alliance for Research in Exercise, Nutrition and Activity Allied Health and Human Performance University of South Australia Adelaide Australia; 2 Melbourne Centre for Behaviour Change School of Psychological Sciences and School of Health Sciences University of Melbourne Melbourne Australia; 3 Alliance for Research in Exercise, Nutrition and Activity Clinical and Health Sciences University of South Australia Adelaide Australia

**Keywords:** virtual assistant, chatbot, Mediterranean diet, physical activity, lifestyle

## Abstract

**Background:**

Poor diet and physical inactivity are leading modifiable causes of death and disease. Advances in artificial intelligence technology present tantalizing opportunities for creating virtual health coaches capable of providing personalized support at scale.

**Objective:**

This proof of concept study aimed to test the feasibility (recruitment and retention) and preliminary efficacy of physical activity and Mediterranean-style dietary intervention (MedLiPal) delivered via artificially intelligent virtual health coach.

**Methods:**

This 12-week single-arm pre-post study took place in Adelaide, Australia, from March to August 2019. Participants were inactive community-dwelling adults aged 45 to 75 years, recruited through news stories, social media posts, and flyers. The program included access to an artificially intelligent chatbot, Paola, who guided participants through a computer-based individualized introductory session, weekly check-ins, and goal setting, and was available 24/7 to answer questions. Participants used a Garmin Vivofit4 tracker to monitor daily steps, a website with educational materials and recipes, and a printed diet and activity log sheet. Primary outcomes included feasibility (based on recruitment and retention) and preliminary efficacy for changing physical activity and diet. Secondary outcomes were body composition (based on height, weight, and waist circumference) and blood pressure.

**Results:**

Over 4 weeks, 99 potential participants registered expressions of interest, with 81 of those screened meeting eligibility criteria. Participants completed a mean of 109.8 (95% CI 1.9-217.7) more minutes of physical activity at week 12 compared with baseline. Mediterranean diet scores increased from a mean of 3.8 out of 14 at baseline, to 9.6 at 12 weeks (mean improvement 5.7 points, 95% CI 4.2-7.3). After 12 weeks, participants lost an average 1.3 kg (95% CI –0.1 to –2.5 kg) and 2.1 cm from their waist circumference (95% CI –3.5 to –0.7 cm). There were no significant changes in blood pressure. Feasibility was excellent in terms of recruitment, retention (90% at 12 weeks), and safety (no adverse events).

**Conclusions:**

An artificially intelligent virtual assistant-led lifestyle-modification intervention was feasible and achieved measurable improvements in physical activity, diet, and body composition at 12 weeks. Future research examining artificially intelligent interventions at scale, and for other health purposes, is warranted.

## Introduction

Poor diet and physical inactivity are amongst the leading modifiable causes of death and disease and increase the risk of developing chronic health conditions such as type 2 diabetes, cardiovascular disease, obesity, cancers, depression, and anxiety [1]. In highly developed countries, these unfavorable health behaviors are pervasive; for example, over ninety percent of Australian adults fail the recommended daily intake of vegetables and fruit, and approximately two-thirds do not meet guidelines of 30 min of physical activity most days [2,3].

Despite the overwhelming benefits of healthy eating and physical activity, making and sustaining lifestyle modification is tremendously challenging [4]. Specialized support from health professionals, such as dietitians and exercise physiologists or physiotherapists, can help people achieve lifestyle change. However, health systems’ finite budgets typically limit access to such services to specific patient populations, such as those with established chronic disease [5]. Consequently, many people with unfavorable lifestyle patterns, who in time will go on to develop chronic disease, do not have access to health professional support to help them modify their lifestyle and prevent disease.

Technology advances in the fields of artificial intelligence and wearables offer new opportunities to deliver accessible, personalized, and cost-efficient behavior modification programs. In particular, virtual assistants use artificial intelligence to mimic human conversation and can be programmed with scripted conversations, questions and answers, and the ability to provide personalized responses based on input from the user. Basic virtual assistants have limited functionality and restrict users’ inputs (eg, by offering questions with multiple-choice response options). For example, weight-loss virtual assistant “Lark” led to significant weight loss, comparable in magnitude to that achieved in in-person lifestyle interventions.[6] However, Lark only allowed users to respond to program-directed questions and did not allow users to ask questions. In contrast, sophisticated virtual assistant technology is capable of recognizing free-written or spoken language (natural language processing), enabling more natural and user-directed communication. Compared to more traditional knowledge transfer methods in digital health (eg, tailored modules), the conversational methods in virtual assistance may be perceived as more personally relevant and humanistic, which is critical given that perceived relevance and the inclusion of social support are associated with increased effect sizes in digital behavior change interventions [7,8].

Preliminary research supports the acceptability and efficacy of natural language virtual assistants in health; however, few studies have been conducted to date. Laranjo and colleagues’ 2018 systematic review of studies evaluating natural language processing virtual assistants in healthcare identified just 14 studies, most of which focused on mental health (n=6) or clinician decision making (n=4) [9]. Single studies (n=3) used virtual assistants to provide patients with education and support for asthma, sexual health, and language impairment [9].

Using an identical search strategy, we updated this review in January 2020 and identified a further 11 studies. Similar to the previous review, most recent studies were aimed at mental health self-management (n=5) [10-12]. Single studies (n=3) used virtual assistants to provide patients with education and support for breast cancer [13], genetic counseling [14], and clinician training [15]. Despite the potential for natural language virtual assistants to provide personalized information and support users to engage in positive health behaviors, only three natural language processing virtual assistants focus on lifestyle modification. “J’arrête de fumer” assisted users in quitting smoking by profiling smoking behavior and providing advice and support at times when cravings were likely to occur [16]. A feasibility study published in 2019 employed a virtual assistant “Tess” to support obese adolescents in health-promoting behavior change with success [17]; however, Tess played a support role only and was designed to supplement ongoing in-person hospital-based services. Additionally, “Reflection Companion” prompted participants to reflect on their physical activity patterns, goals, barriers, and enablers of activity but was unable to provide positive affirmation or encouragement, answer participants’ questions, or provide tailored advice [18].

In sum, although the use of virtual assistants in healthcare is a rapidly developing field, few virtual assistants use natural language processing to promote lifestyle change and those that do remain basic, performing limited, highly specific processes. To our knowledge, there are currently no published studies examining fully automated, natural language processing artificial intelligence health coaches for lifestyle modification.

This proof-of-concept study aimed to assess the feasibility of an artificial intelligence virtual health coach-led physical activity and diet intervention for community-dwelling middle-aged and older adults (recruitment and retention) and to evaluate the preliminary efficacy of the virtual health coach intervention for improving physical activity, Mediterranean-style diet adherence, and health risk factors.

## Methods

The case-controlled study was approved by the University of South Australia Human Research Ethics Committee (ref 201724). All participants provided informed consent before commencing the study.

### Intervention

The 12-week MedLiPal program assists users in increasing their lifestyle physical activity and adopt a Mediterranean-style diet. It incorporates numerous behavior change techniques to target determinants of lifestyle behaviors, including goal-setting, problem-solving, goal review, self-monitoring with feedback, social support, reattribution, and use of credible sources [19]. The components include Paola, a wearable activity monitor, the MedLiPal website, and a diet and activity log.

**Figure 1:**
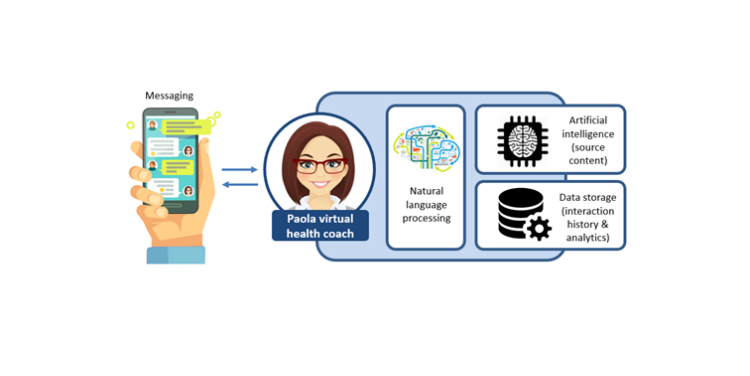
Overview of the technology underpinning Paola, the virtual health coach.

‘Paola’ is a natural language processing virtual health coach (Figure 1), created using IBM Watson Virtual Assistant artificial intelligence software (IBM Corp.). Paola performs three key roles: (1) guides participants through an introductory session during which she teaches them about the principles of physical activity, goal-setting and self-monitoring, and the Mediterranean diet, including recommended daily and weekly servings (based on Davis protocol) [20]; (2) guides users through 11 weekly check-ins regarding their daily steps and dietary pattern for the past week, and assists users to set personalized step and diet goals for the coming week; and (3) is available 24/7 to answer users’ questions. Paola was deployed on the cloud-based instant messaging platform Slack (Slack Technologies). Participants were required to download the Slack application on their device and create a user account to access Paola.

Paola referred to users by their first name and could respond to questions at any time. Frequently asked questions (eg, “How much bread can I eat?”) were categorized by the *intent* (eg, “how much” type questions) and by the *entity* (eg, breads and cereals). The natural language processing artificial intelligence software underpinning Paola identified intents and entities and selected an appropriate response from stored dialogue options (eg, “You can have up to 5 servings of breads and cereals per day”). Intents, entities, and dialogue selections were developed through collaboration between the health scientists on the study team and the software developers.

We provided participants with a Garmin Vívofit4 wearable activity monitor, which allows users to monitor their daily physical activity levels (Garmin). During the introductory session, Paola assisted participants to set a personal daily step count goal based on age-based normative values +2000 steps, considering their current activity levels. This daily step goal was revisited and edited at each weekly check-in.

The MedLiPal website housed educational videos, study information, and useful links. During the baseline session, Paola linked participants to the website from Slack, requiring them to log in and view the educational videos. Participants could then visit the website for information and recipes at any time. The diet and activity log was used to record daily steps and dietary servings (Multimedia Appendix 1) [20,21]. Details of the Mediterranean diet recommendations are included as Multimedia Appendix 2.

### Participants

Volunteers were recruited during February and March 2019 through mainstream news items, flyers, and social media. Participants were eligible if they were aged 45-75 years, owned an iOS or Android smartphone or tablet with internet access, were not currently meeting the Australian physical activity guidelines, and were not following a Mediterranean dietary pattern. Participants completed an online screening survey, followed by a brief phone interview to confirm eligibility, answer any potential questions participants may have had, and arrange their baseline appointment. Participants’ physical activity level was determined based on a single question enquiring whether they did more than 30 minutes of activity on five or more days per week, while Mediterranean diet pattern was determined using the 14-item Mediterranean diet questionnaire, with a score of 7 or less indicating they were not following the Mediterranean diet [22]. Participants were excluded if they were unable to consume a Mediterranean diet due to allergy or other food aversions, had a medical condition precluding them from increasing their physical activity or were pregnant or lactating. All participants read the information sheet and were provided an opportunity to ask questions before providing informed consent and commencing the study.

### Procedure

The primary outcomes were total minutes of weekly moderate-to-vigorous physical activity and Mediterranean diet adherence, measured via an online survey at baseline, week 6, and week 12. Total minutes of weekly moderate to vigorous physical activity was assessed using the Active Australia Survey (AAS) [23,24]. Mediterranean diet adherence was measured using a 14-item Australian Mediterranean diet adherence tool, adapted from the Prevención con Dieta Mediterránea (PREDIMED) study [25] to align with the Australian food supply [26]. The Australian Mediterranean diet adherence tool has been validated relative to the Mediterranean diet score calculated from a 3-day weighed food record r=0.44 [26].

Participants attended in-person assessments at baseline, 6 weeks, and 12 weeks for secondary outcome measures. Secondary outcomes included: body weight (Seca 703), measured while clothed and with shoes removed; height measured without shoes (Seca 206); waist circumference, measured at the midpoint between the pelvic crest and bottom rib, unless a visual narrow was present elsewhere (Lufkin Thinline 2 mm, Apex Tool Group) [27]; systolic and diastolic blood pressure (Omron Healthcare) [26].

Sociodemographic characteristics (sex, age) and medication use were captured in the online survey. Socioeconomic status was measured according to the Socio-Economic Index for Areas disadvantage index, based on postcode [28].

Following the completion of the baseline assessments, participants were provided with a Garmin Vívofit4 activity tracker and access to the virtual health coach and the MedLiPal website. Initial login prompted Paola to begin the introductory session, in which the health coach introduced herself and taught users about the Mediterranean lifestyle and its benefits, the principles of increasing physical activity, goal-setting, and how to use the Garmin Vívofit4 activity tracker, the Mediterranean dietary pattern, and how to self-monitor daily and weekly servings using the MedLiPal weekly log sheet. Paola then invited the participant to converse with her regarding any questions they had about the program. Paola also explained to participants that she was available 24/7 to answer their questions.

Each week, users received an email notification inviting them to complete a weekly check-in with Paola to check their progress, assisting them with setting new weekly goals and answering their questions.

Participants did not receive an honorarium but could keep their Garmin Vívofit4 activity tracker after the study.

### Feasibility

Feasibility was judged based on recruitment, retention, and engagement. We sought to recruit 30 participants to the study within 6 weeks, on the rationale that if this could be achieved with no advertising budget, a future definitive trial with a dedicated recruitment budget should be able to recruit approximately 400 participants over a year. We set a retention threshold of 75% at 12 weeks. Behavioral engagement with the MedLiPal program was assessed via virtual health coach usage data (number of weekly check-ins completed). The a priori engagement target was set at 70% (ie, that participants would complete at least 8 of 11 weekly sessions with Paola).

### Power and Statistical Analyses

The target sample size was set at n=30, which was considered adequate to answer feasibility research questions. For efficacy outcomes, this sample size, assuming 80% power, an alpha of 0·05, and a single group design with three repeated measures, was able to detect an effect size of *d*=0.48.

Feasibility data and participants’ sociodemographic characteristics were analyzed descriptively, using means and standard deviations, frequencies, and percentages. Efficacy was assessed using an unadjusted repeated measures ANOVA, conducted on an intention-to-treat principle. Given that a small amount of data was missing (16% missing data at week 6, 10% at week 12 for primary outcomes), imputation was achieved using the last observation carried forward. Sensitivity analyses using complete cases were also conducted. All analyses were performed using SPSS (version 26, IBM) with a *P* value of .05. Pairwise comparisons included Bonferroni adjustment, and exact *P* values are reported.

## Results

### Recruitment and Retention

**Figure 2:**
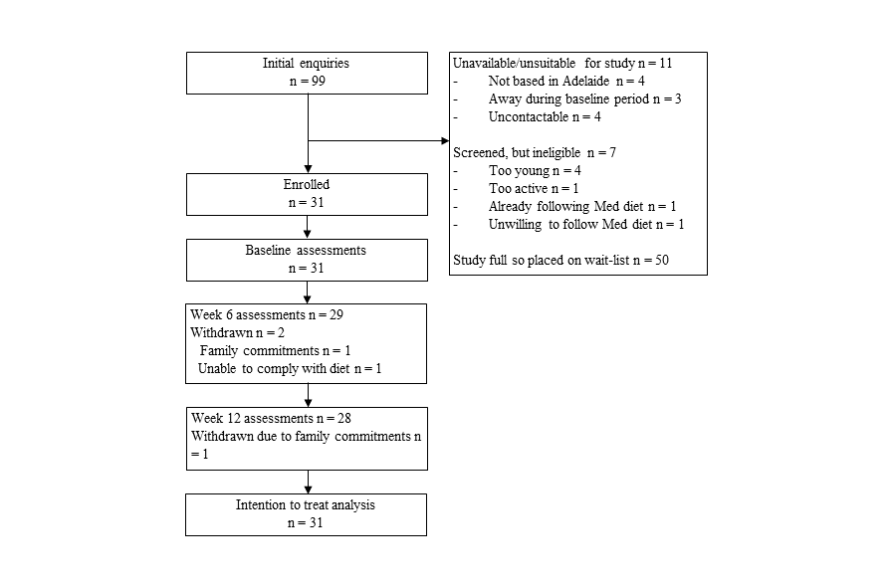
Participant flow through the study.

A total of 99 potential participants formally expressed their interest in the study within four weeks of recruitment opening, at which point expressions of interest were closed. The first 38 potential participants completed the eligibility screening interview, of which 31 (82%) were eligible. Participants were sequentially booked for baseline assessments until the study quota of 30 participants was filled, with n=31 ultimately enrolled. Attendance for the 6- and 12-week assessments was 29 and 28, respectively (Figure 2).

### Participant Characteristics

Participants’ baseline demographic and clinical characteristics are provided in Table 1. On average, participants were aged 56.2 years (SD 8.0), and two-thirds were women. Most participants were either overweight or obese.

**Table 1:** Baseline characteristics of participants in the MedLiPal study.

		Men (n=10)	Women (n=21)	All (n=31)
Age (years), mean (SD)		60.6 (8.7)	54.1 (7.0)	56.2 (8.0)
**BMI, n (%)**				
	Underweight	0 (0)	0 (0)	0 (0)
	Normal	1 (10)	7 (33)	8 (26)
	Overweight	4 (40)	6 (29)	10 (32)
	Obese	5 (50)	8 (38)	13 (42)
Weekly MVPA^a^ (minutes), mean (SD)		308.7 (172.2)	157.2 (143.0)	206.1 (166.5)
Mediterranean diet adherence score (out of 14), mean (SD)		3.9 (2.1)	3.8 (1.6)	3.8 (1.8)
Waist circumference (cm), mean (SD)		102.4 (8.5)	93.0 (18.0)	96.0 (16.0)
SBP (mmHg)^b^, mean (SD)		135.8 (11.1)	120.8 (10.6)	125.7 (12.8)
DBP (mmHg)^c^, mean (SD)		85.3 (11.2)	81.6 (5.7)	82.8 (7.9)
Socioeconomic status^d^, mean (SD)		997.4 (62.5)	1001.9 (64.3)	1000.5 (62.8)

^a^MVPA: moderate and vigorous physical activity.

^b^SBP: systolic blood pressure.

^c^DBP: diastolic blood pressure.

^d^Socio-Economic Index for Areas (SEIFA) national mean = 1000±100.

### Behavioral and Health Outcomes

The efficacy results are shown in Table 2. From baseline to week 6, weekly physical activity increased by approximately one hour, and then by a further 50 minutes at 12 weeks. Thus overall, from baseline to 12 weeks, physical activity increased by 109.8 minutes (95% CI 1.9 to 217.7, *P*=.005). Mediterranean diet adherence increased markedly from baseline to week 6 and then was approximately maintained at this level from week 6 to week 12 (mean change baseline to 12 weeks 5.7, 95% CI 4.2 to 7.3, *P*<.001).

On average, participants lost 1.1 kg from baseline to week 6, and then lost a further 0.2 kg to week 12, resulting in an overall average loss of 1.3 kg (95% CI –2·5 to –0·7, *P*=.01). Similarly, waist circumference decreased by 1 cm from baseline to week 6, and then another 1 cm to week 12, leading to an overall loss of –2.1cm (95% CI –3.5 to –0.7, *P*=.003). There was no change in blood pressure (diastolic or systolic) at either time point.

Sensitivity analyses (Multimedia Appendix 3) were conducted using complete case data (n=28). Results were consistent with the intention-to-treat (ITT) analyses, though effect sizes tended to be slightly larger; for example, physical activity increased by 145 minutes (vs 110 minutes in the ITT analyses) and that Mediterranean diet adherence score increased by 6.5 points (vs 5.7 points in the ITT analysis).

**Table 2:** Outcome measures at baseline, 6 weeks, and 12 weeks.

Outcome measure	Baseline	6 weeks	12 weeks	*F (2, 29)*, *P* for overall model	Difference from baseline to Week 6 (95% CI)^a^	Difference from baseline to Week 12 (95% CI)^a^
	Mean (SD)			Mean (SD)	Mean (SD)	
Weekly total MVPA^b^ minutes (min)	206.1 (166.5)	266.8 (207.2)	315.9 (261.7)	6.45, .005	60.8 (–21.7 to 143.3)	109.8 (1.9 to 217.7)
Mediterranean diet adherence score (out of 14)	3.8 (1.8)	9.8 (3.7)	9.6 (3.1)	44.56, <.001	6.0 (4.3 to 7.7)	5.7 (4.2 to 7.3)
Weight (kg)	83.6 (19.0)	82.4 (18.3)	82.3 (18.1)	5.41,.01	–1.1 (–2.0 to –0.3)	–1.3 (–2.5 to –0.1)
Waist circumference (cm)	96.0 (16.0)	95.1 (15.8)	93.9 (15.8)	7.13,.003	–1.0 (–1.8 to –0.1)	–2.1 (–3.5 to –0.7)
SBP^c^ (mmHg)	125.7 (12.8)	124.9 (14.5)	125.5 (13.8)	0.11,.90	–0.8 (–4.9 to 3.4)	–0.2 (–5.7 to 5.3)
DBP^d^ (mmHg)	82.8 (7.9)	81.8 (9.3)	81.8 (8.8)	0.64, .54	–1.0 (–3.4 to 1.3)	–1.0 (–4.3 to 2.4)

^a^Pairwise comparisons confidence intervals include Bonferroni adjustment for multiple comparisons.

^b^MVPA: moderate and vigorous physical activity.

^c^SBP: systolic blood pressure.

^d^DBP: diastolic blood pressure.

### Engagement With the Virtual Health Coach

**Figure 3:**
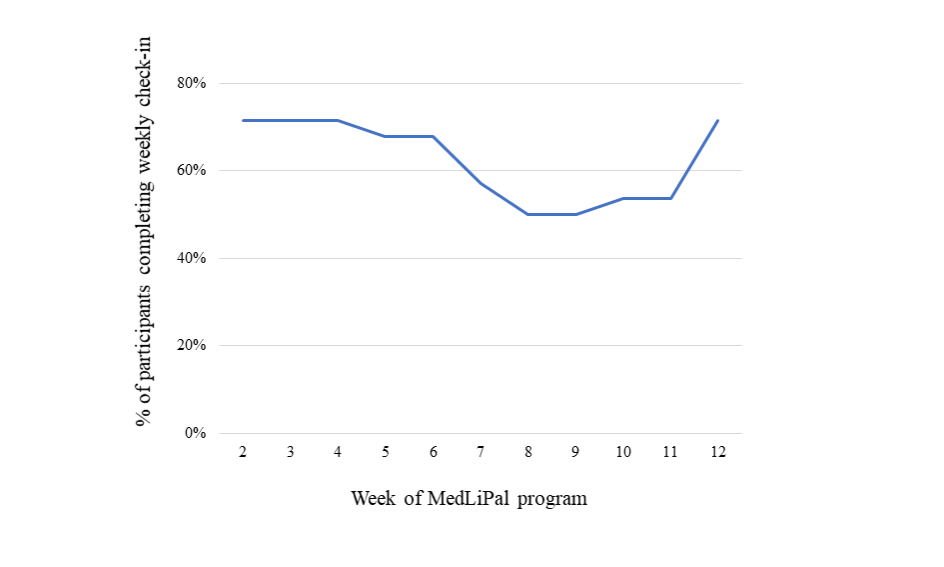
The percentage of participants completing the weekly virtual health coach check-in across the 12-week intervention period.

From Weeks 2-12 of the program, participants were encouraged to complete weekly check-ins with Paola, the virtual health coach. Out of a maximum of 11 possible check-ins, participants completed an average of 6.9 (64%, range 1-11). Engagement with the check-ins across the intervention period is shown in Figure 3. Around 70% of participants completed the check-ins in weeks two, three, four and 12, with engagement gradually sliding to around 50% through weeks eight and nine, before rising again in the final weeks of the program. Participants who completed the first weekly check-in had significantly higher engagement across the intervention period than those who did not complete the first weekly check-in (mean 7.4, SD 3.4 vs mean 4.6, SD 2.6; *P*=.03).

### Other Feasibility Issues

Of the 31 participants meeting all eligibility criteria and enrolled in the study, it became clear during baseline appointments that 3 participants had minimal smartphone skills (eg, did not know how to send a text message or download an app). These participants were reliant on their partners or children to use their phones as part of the study. At the 6-week assessment, several participants reported difficultly consuming the recommended number of food group servings (due to feeling satiated). They were encouraged to ask Paola for advice. Paola’s advice to users reporting satiation was to reduce portion sizes across all food groups and to maintain a variety of healthy foods

### Adverse Events

No adverse events related to study participation were experienced.

## Discussion

### Principal Results

This study aimed to assess the feasibility of a highly innovative virtual health coach physical activity and diet intervention for middle-aged and older adults. The study found a high level of interest in the technology-based intervention approach amongst the target demographic and high retention across the 12-week study period. Preliminary evidence suggests that the intervention led to sizable improvements in physical activity, diet, and body composition across 12 weeks.

The intervention approach was well-received by participants, exceeding our a priori expectations for recruitment and retention. Engagement with the virtual health coach (based on completed weekly check-ins) was slightly lower than our a priori threshold of ≥70% (actual completion rate 64%). Interestingly, the most significant drop off with the virtual health coach occurred between the baseline appointment and week two of the program (ie, the first check-in participants were supposed to complete on their own), with around a quarter of participants failing to complete the first weekly check-in. Three participants who enrolled in the study were found to have very low smartphone literacy at the baseline session, which impacted their ability to use the program as intended. In some cases, technical problems with Slack or the software interrupted attempts to check in, which generally were rectified after week 1. It appears that getting participants to complete their first independent check-in successfully may be a strong predictor of ongoing engagement, and a specific target in future program iterations. It may be useful to provide participants with a courtesy follow-up phone call after the first week to ensure they were able to complete the first check-in and proactively troubleshoot issues. Allowances for low smartphone literacy could be addressed in the study protocol, for example, by offering in-person assistance and written step-by-step instructions and troubleshooting information.

Our study attracted mostly women, who were similar to national averages for socioeconomic advantage (national average 1000) [28]. Our sample had poor adherence to Australian healthy eating guidelines (baseline average self-reported vegetable servings was 2.2/day and fruit 1.3/day compared to the recommended 5 and 2 servings per day, respectively) [29]. They also exhibited above-average rates of obesity and overweight compared with population norms [30] and physical activity rates comparable to other Australian studies of older adults measured using the AAS [31-33]. This population appears to be ideally suited to this type of new offering, given that they are not unwell enough to qualify for face-to-face services through the health system, yet have modifiable risk factors for the future development of chronic diseases such as type 2 diabetes and cardiovascular disease.

Feasibility data collected in the study are invaluable to inform future trials, which could take the form of a traditional randomized controlled trial to determine the efficacy of the current intervention package or utilize a modern research design, such as the micro-randomization trial design, which allows multiple intervention elements to be experimentally evaluated [34]. The latter is useful for optimizing intervention packages for future use. Results suggest there is considerable enthusiasm amongst older adults for this style of intervention and confirm that most older adults have sufficient skills to access a virtual health coach program and use it effectively to achieve sizeable behavior and health change. Future research may also focus on thoroughly understanding the user experience of virtual health coach programs, which would be useful to inform improvements, for example, in terms of the virtual health coach’s language style, variety of language, and possibly, sense of humor (which improves the humanity and emotional connection of artificial-intelligence virtual assistants [35]). Given the rapid technological advancements occurring in this field, there are many other opportunities for enhancing this intervention to address poor health behaviors, which may allow targeting of specific health issues, such as weight loss or diabetes self-management.

### Comparison With Prior Work

To our knowledge, this is the first study to evaluate the capacity of an advanced virtual assistant technology to deliver a personalized lifestyle intervention amongst older adults. The magnitude of change in physical activity was similar to [36,37] or larger than [38,39] that achieved in previous computer-tailoring interventions. Improvement in Mediterranean diet adherence was much more significant than those achieved by previous technology-based Mediterranean diet interventions [40,41]. The degree of behavior change achieved was approximately in line with that reported in previous studies using intensive one-on-one dietary counseling [26,42]. These exciting findings suggest that artificial intelligence technology may be able to provide practical, supportive dietary counseling at a low cost.

### Limitations

This study’s primary strength is its ground-breaking intervention approach of using an artificially intelligent virtual health coach to deliver a personalized physical activity and dietary intervention program. Our virtual health coach used natural language processing, which allows users to converse using freely written language, representing a significant advancement over previous computer-tailoring approaches, which typically rely on multiple choice. Other study strengths include its mostly hands-off approach (thus improving the ecological validity of the study compared with a study with a high level of human contact), the use of objective health measures, and the use of intention-to-treat analysis, which ensures that the intervention effects are not over-estimated. Limitations include the pre-post design, which meant the study lacked a randomized control comparator, and follow-up is limited to three months.

### Conclusions

Virtual assistant technology offers exciting potential for the delivery of highly personalized, scalable health interventions. They can make evidence-based advice and support available to patients who are not unwell enough to quality for in-person (ie, expensive) services. They also have the potential to be used as adjunct support for those patients with complex and chronic health conditions such as type 2 diabetes or celiac disease, who see a health practitioner, but would benefit from additional support. Furthermore, they present a way of providing a scientifically substantiated dietary pattern and lifestyle programs to rural or remote areas or isolated individuals.

This ground-breaking study confirmed that a virtual health coach diet and physical activity intervention is feasible for older adults and leads to sizable health behavior change and improvements in body composition. Future research examining artificially intelligent interventions at scale, and for other health purposes, is warranted.

#### Availability of Data and Materials

The datasets used and analyzed for this study are available from the corresponding author on request.

## Appendix

Multimedia Appendix 1 Physical activity and diet log sheet, for recording daily steps and dietary intake, adapted from Davis et al.

Multimedia Appendix 2 Specific recommendations for the Mediterranean diet, based on MedLey diet.

Multimedia Appendix 3 Outcome measures at baseline, 6 weeks and 12 weeks – complete case analysis (n=28).

## Abbreviations

AAS: Active Australia Survey
MVPA: moderate and vigorous physical activity
PREDIMED: Prevención con dieta Mediterránea
SBP: systolic blood pressure
DBP: diastolic blood pressure
MedLiPal: Mediterranean lifestyle and physical activity
