# Supplementary material for: A Physical Activity and Diet Program Delivered by Artificially Intelligent Virtual Health Coach: Proof-of-Concept Study
Source: JMIR Mhealth Uhealth. 2020 Jul 10;8(7):e17558. doi: 10.2196/17558 (PMC7382010; doi:10.2196/17558)
Supplement: Multimedia Appendix 1 [file mhealth_v8i7e17558_app1.docx]

**Supplementary file 1. Physical activity and diet log sheet, for recording daily steps and dietary intake, adapted from Davis et al^1^.**

1 Davis C, Hodgson J, Bryan J, Garg M, Woodman R, Murphy K. Older Australians can achieve high adherence to the Mediterranean diet during a 6-month randomised intervention; results from the Medley study. *Nutrients* 2017; **9**(6): 534, DOI: 10.3390/nu9060534.

| **Participant ID:**  ***Week#: _________*  Write in your daily step counts, and mark when you’ve had a serving of food** | | | | | | | | | |
| --- | --- | --- | --- | --- | --- | --- | --- | --- | --- |
|  | **This weeks’ daily STEP GOAL ________** | **DAY 1**  ***Date:*** | **DAY 2**  ***Date:*** | **DAY 3**  ***Date:*** | **DAY 4**  ***Date:*** | **DAY 5**  ***Date:*** | **DAY 6**  ***Date:*** | **DAY 7**  ***Date:*** | **Week average** |
|  | **Total step count:** |  |  |  |  |  |  |  |  |
| **Serving size** | **FOODS TO EAT DAILY** |  |  |  |  |  |  |  | **Week total** |
| 1 cup salad  ½ cup cooked | **Vegetables:** 5 or more serves per day |  |  |  |  |  |  |  |  |
| 1 Tbsp. | **Extra Virgin Olive Oil:** 2-4 Tbsp. per day |  |  |  |  |  |  |  |  |
| 1 pc, 150 g | **Fruit:** 2-3 serves per day |  |  |  |  |  |  |  |  |
| 1 slice bread  1/2 cup pasta  2/3 cup cereal | **Grain foods:** (bread, pasta, cereal, rice) 5 serves per day |  |  |  |  |  |  |  |  |
| 1 cup milk  ¾ cup yog  40 g cheese | **Dairy foods:** 2 serves per day |  |  |  |  |  |  |  |  |
| 100 ml | **Red wine:** 200 ml per day or less |  |  |  |  |  |  |  |  |
|  | **FOODS TO EAT WEEKLY** | **DAY 1** | **DAY 2** | **DAY 3** | **DAY 4** | **DAY 5** | **DAY 6** | **DAY 7** | **Week total** |
| 75 g | **Legumes:** 3 serves or more per week |  |  |  |  |  |  |  |  |
| 30 g | **Nuts:** 5 serves or more per week |  |  |  |  |  |  |  |  |
| 150 g raw | **Fish:** 3 serves or more per week |  |  |  |  |  |  |  |  |
| Olive oil, tomato, onion, garlic sautéed | **Sofrito sauce:** 2 times per week or more |  |  |  |  |  |  |  |  |
| 100 g raw | **Chicken/poultry:** no more than 2 serves per week |  |  |  |  |  |  |  |  |
| 100 g raw | **Red meat:** no more than 1 serve per week |  |  |  |  |  |  |  |  |
| 1 egg (60 g) | **Eggs:** 6 or less per week |  |  |  |  |  |  |  |  |
| 600 kJ/150 kcal | **Discretionary foods:** (chocolate, lollies, cake) |  |  |  |  |  |  |  |  |
