# Supplementary material for: A Physical Activity and Diet Program Delivered by Artificially Intelligent Virtual Health Coach: Proof-of-Concept Study
Source: JMIR Mhealth Uhealth. 2020 Jul 10;8(7):e17558. doi: 10.2196/17558 (PMC7382010; doi:10.2196/17558)
Supplement: Multimedia Appendix 2 [file mhealth_v8i7e17558_app2.docx]

**Supplementary file 2: Specific recommendations for the Mediterranean diet, based on MedLey diet^1^**

| **Food group** | **Recommendation** |
| --- | --- |
| **Daily** | |
| 1. Olive oil, extra virgin | 36-72 ml, 2-4 Tbsp |
| 1. Vegetables | ≥400 g, approximately 5-6 serves, including leafy greens |
| 1. Fruit | 2-3 serves, 300-450 g fresh fruit, or equivalent canned/dried fruit |
| 1. Grain foods | 5 serves, choose wholegrain |
| 1. Red wine | ≤200 ml, with meals |
| 1. Dairy | 2 serves, mostly as cheese and yoghurt |
| **Weekly** | |
| 1. Sofrito sauce | 2/week, onions, garlic and tomato sautéed in olive oil (combined with pasta, salad, meat, vegetables, pizza) |
| 1. Legumes | 250 g, approximately 2.5 cups or 3-4 serves |
| 1. Fish | 300-400 g, include at least one serve oily fish |
| 1. Red meat | ≤100 g |
| 1. Chicken/poultry | 100-200 g |
| 1. Smallgoods | ≤100 g |
| 1. Nuts | ≥5 serves, approximately 30 g or 1 handful |
| 1. Eggs | ≤6-7 |
| 1. Sweets, processed foods | ≤3 times, in small portions (e.g. small slice cake) |

1 Davis CR, Bryan J, Hodgson JM, Wilson C, Dhillon V, Murphy KJ. A randomised controlled intervention trial evaluating the efficacy of an Australianised Mediterranean diet compared to the habitual Australian diet on cognitive function, psychological wellbeing and cardiovascular health in healthy older adults (MedLey study): protocol paper. *BMC Nutr* 2015; **1**(1): 35.
