# Supplementary material for: A Physical Activity and Diet Program Delivered by Artificially Intelligent Virtual Health Coach: Proof-of-Concept Study
Source: JMIR Mhealth Uhealth. 2020 Jul 10;8(7):e17558. doi: 10.2196/17558 (PMC7382010; doi:10.2196/17558)
Supplement: Multimedia Appendix 3 [file mhealth_v8i7e17558_app3.docx]

| Outcome measure | Baseline  (mean (SD)) | 6 weeks  (mean (SD)) | 12 weeks  (mean (SD)) | F, p | Difference from baseline to Week 6 (95% CI)* | Difference from baseline to Week 12 (95% CI)* |
| --- | --- | --- | --- | --- | --- | --- |
| Weekly total MVPA minutes (min) | 202·0 (180·5) | 284·5 (226·2) | 316·6 (246·3) | 6·83, 0·005 | 82·5 (-23·6 to 188·5) | 144·5 (-8·8 to 237·8) |
| Mediterranean diet adherence score (out of 12) | 4·0 (1·9) | 11·2 (2·0) | 10·5 (2·1) | 80·60, p<0·001 | 7·2 (5·8 to 8·6) | 6·5 (5·0 to 8·0) |
| Weight (kg) | 80·8 (16·7) | 79·8 (16·3) | 79·6 (16·0) | 5·53, 0·01 | -1·0 (-1·8 to -0·2) | -1·2 (-2·4 to 0·1) |
| Waist circumference (cm) | 94·0 (15·1) | 93·0 (14·9) | 91·8 (14·7) | 6.80, 0·004 | -1·0 (-1·9 to 0·04) | -2·2 (-3·8 to -0·7) |
| SBP (mmHg) | 125·5 (12·9) | 125·1 (15·2) | 125·7 (14·4) | 0·05, 0·96 | -0·4 (-4·9 to 4·1) | 0·2 (-5·8 to 6·2) |
| DBP (mmHg) | 82·4 (7·9) | 81·3 (9·5) | 81·3 (9·0) | 0·58, 0·57 | -1·1 (-3·8 to 1·6) | -1·0 (-4·8 to 2·7) |
| *Pairwise comparisons confidence intervals include Bonferroni adjustment for multiple comparisons. | | | | | | |

**Supplementary file 3. Outcome measures at baseline, 6 weeks and 12 weeks – complete case analysis (n=28).**
